# Modeling and affinity maturation of an anti-CD20 nanobody: a comprehensive *in-silico* investigation

**DOI:** 10.1038/s41598-023-27926-4

**Published:** 2023-01-11

**Authors:** Alireza Poustforoosh, Sanaz Faramarz, Manica Negahdaripour, Hassan Hashemipour

**Affiliations:** 1 Department of Chemical Engineering, Faculty of Engineering, Shahid Bahonar University of Kerman, Kerman, Iran; 2 Medicinal and Natural Products Chemistry Research Center, Shiraz University of Medical Sciences, Shiraz, Iran; 3 Department of Clinical Biochemistry, Afzalipour School of Medicine, Kerman University of Medical Sciences, Kerman, Iran; 4 Department of Pharmaceutical Biotechnology, School of Pharmacy, Shiraz University of Medical Sciences, Shiraz, Iran; 5 Pharmaceutical Sciences Research Center, School of Pharmacy, Shiraz University of Medical Sciences, Shiraz, Iran; 6 Chemical Engineering Department, Faculty of Engineering, Vali-e-Asr University of Rafsanjan, Rafsanjan, Iran

**Keywords:** Biophysical chemistry, Structural biology, Cancer, Computational biology and bioinformatics, Drug discovery

## Abstract

B-cell Non-Hodgkin lymphomas are the malignancies of lymphocytes. CD20 is a membrane protein, which is highly expressed on the cell surface of the B-cells in NHL. Treatments using monoclonal antibodies (mAbs) have resulted in failure in some cases. Nanobodies (NBs), single-domain antibodies with low molecular weights and a high specificity in antigen recognition, could be practical alternatives for traditional mAbs with superior characteristics. To design an optimized NB as a candidate CD20 inhibitor with raised binding affinity to CD20, the structure of anti-CD20 NB was optimized to selectively target CD20. The 3D structure of the NB was constructed based on the optimal templates (6C5W and 5JQH), and the key residues were determined by applying a molecular docking study. After identifying the key residues, some mutations were introduced using a rational protocol to improve the binding affinity of the NB to CD20. The rational mutations were conducted using the experimental design (Taguchi method). Six residues (Ser27, Thr28, Phe29, Ile31, Asp99, and Asn100) were selected as the key residues, and five residues were targeted for rational mutation (Trp, Phe, His, Asp, and Tyr). Based on the mutations suggested by the experimental design, two optimized NB structures were constructed. NB2 showed a remarkable binding affinity to CD20 in docking studies with a binding energy of − 853 kcal/mol. The optimized NB was further evaluated using molecular dynamics simulation. The results revealed that CDR1 (complementarity determining regions1) and CDR3 are essential loops for recognizing the antigen. NB2 could be considered as a potential inhibitor of CD20, though experimental evaluations are needed to confirm it.

## Introduction

Non-Hodgkin lymphomas (NHL) are heterogeneous malignant diseases originating from lymphocytes. They could be initiated at different steps of differentiation^1^. About 85–90% of the NHL originates from B cells^2^. One of the ideal targets for the treatment of B-cell non-Hodgkin lymphomas (B-NHL) is CD20, since it has specific biological characteristics and pattern of expression^3^. Rituximab is an approved therapy for B-NHL (since 1997), which has increased the patients survival along with acceptable toxicity^4,5^. In spite of the success achieved by rituximab in B-NHL treatment, there are some patients who fail to respond to the initial therapy^6^. Therefore, there is an urgent demand to develop an effective treatment for B-NHL that can inhibit CD20. An alternative strategy to overcome this problem is using monoclonal antibodies (mAbs) against CD20^7^.

mAbs havesome limitations that reduce their effectiveness. For instance, the large size of mAbs, such as rituximab (143.86 kDa), decreases their tumor penetration. This problem can lead to the slow distribution and inadequate effectiveness of the cancer treatment^8^. Nanobodies (Nbs) or heavy-chain variable domains (VHHs) are alternative for mAbs that can address such limitations^9^. Nbs are single-domain antibodies derived from camelids and sharks, which have a high sequence identity with the human VH gene family III^10^. Nbs have a single antigen-binding variable region, which is highly stable and soluble because of their low molecular weight (MW) compared to antibodies^11,12^. Their MW is usually less than 15 kDa, making it possible for them to penetrate the target site more easily than conventional antibodies^13^. Some notable features of the NBs, such as thermostability, high binding specificity, and low immunogenicity, make them attractive biological materials for medicinal applications^14^. In addition, NBs posse extended loops of complementarity determining regions (CDR), which enable them to bind to a broader range of epitopes. The low MW of the NBs and their similarity to the human immunoglobulin variable domain lead to negligible immune response, which means humanization would not be often necessary.

Computational methods are versatile strategies that can provide valuable data about biological systems at various stages^15–17^. These methods can be used for vaccine design^18^ and predict the immunogenicity of biomolecules^19^. The proteomics approach could be used as a practical method to develop drug-like molecules with specific targets^20^. Computational screening techniques have contributed significantly to the development and design of antibodies^21,22^. Some available methods are usually conducted for this purpose. One approach is to perform screening using a known NB that has been found in experimental or computational studies. The experimental methods utilize phage display, bacterial display, yeast display, and ribosome display to develop the desired NB. The computational methods are used to perform docking studies to evaluate the binding affinity of the NBs to the target.

Moreover, the mutation of residues on various parts of the NB, such as CDR or other regions, could provide an optimized version of the original NB^23,24^. The location of CDR or non-CDR loops has to be determined at the first step in these methods. There are available tools for determining and counting CDRs^25^. After recognizing the NB orientation, the mutations could be performed to increase the binding affinity between the NB and the antigen. Recent computational strategies are founded on locating the CDR and non-CDR residues that create interaction with the antigen and accomplishing point mutations to improve the interactions^26–29^. However, specific criteria for the selection of the candidate residues for mutation have not been introduced so far^30^.

The optimized strategy developed here is based on determination of hyper-available residues of the original NB and introduction of rational mutations by performing the experimental design. The residue positions could be determined by performing the molecular docking study and obtaining the detailed results of interactions. After finding adequate residues, an appropriate strategy to apply rational mutations is needed. Design of experiment (DOE) is a useful method for selecting a certain number of experiments instead of a large number of investigations.

The objective of this study is to design an optimized NB with raised binding affinity to CD20, which could be a potential inhibitor candidate of CD20, using the mentioned computational protocol and rational mutations.

## Materials and methods

### Homology modeling

**Figure 1:**
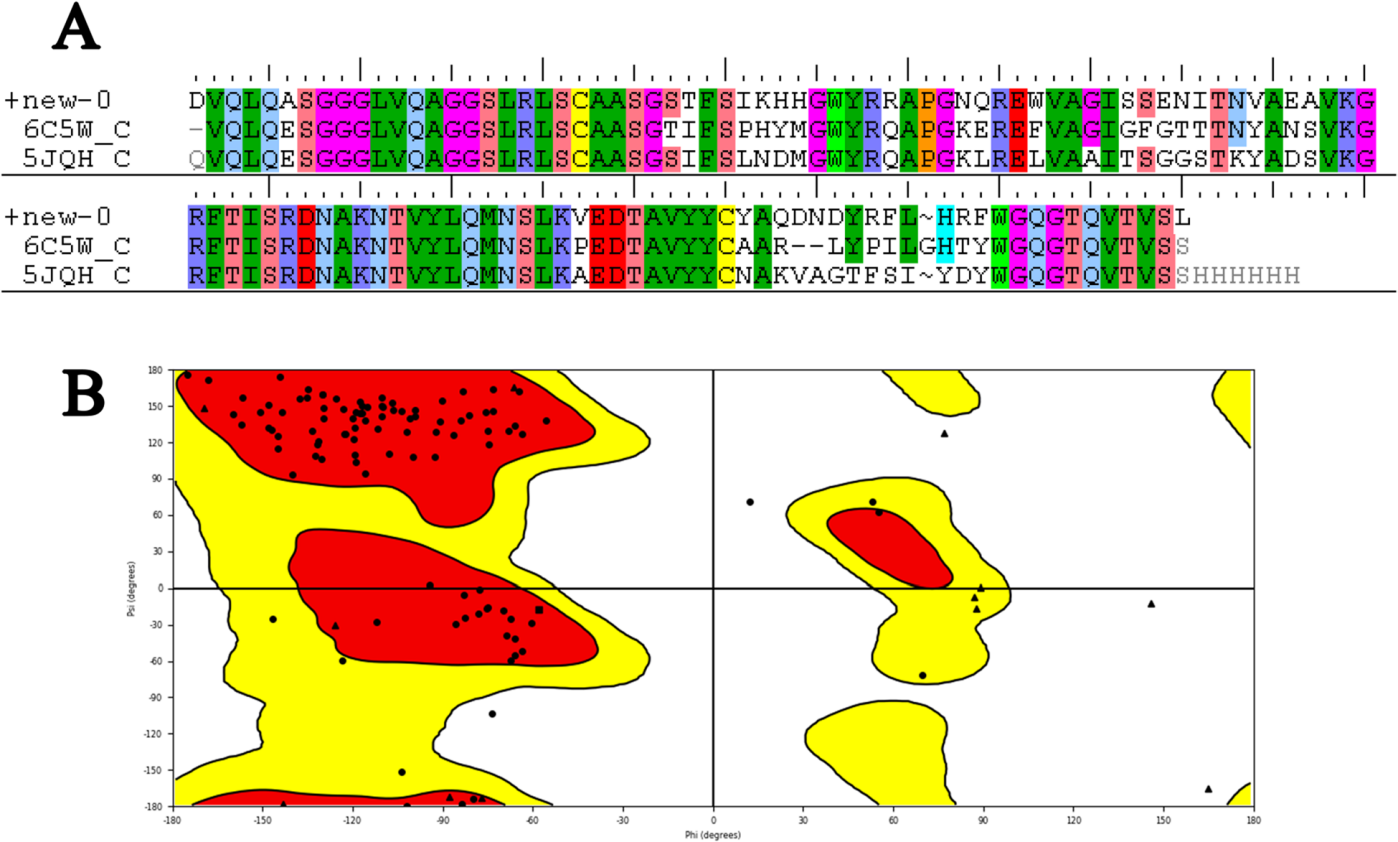
Sequence alignment of nanobody (+ new) and the templates (6C5W and 5JQH) (**A**). The Ramachandran plot of the built NB using the templates (**B**). Red and yellow regions are the most-favored regions and allowed regions, respectively.

The sequences of the nanobody (NB) that binds to human CD20 were obtained from (INDI, http://naturalantibody.com/nanobodies)^31^ (patent number: US20180079822). The sequences are shown in Fig. 1. The BioLuminate of Schrödinger suites was used for homology modeling. Firstly, the BLAST homology search was utilized to find similar structures from the NCBI database. The optimal templates were chosen to construct the 3D model of the NB. The knowledge-based approach was employed for model building. This method utilizes a database derived from all antibody structures presently known in the PDB^32^. The quality of the built model was assessed using the Ramachandran plot, and the conformation of the amino acids backbone was evaluated.

### Molecular docking study

The structures of templates and CD20 (PDB ID: 3PP4)^33^ were obtained from the PDB database (http://www.rcsb.org/pdb), and the preparation of the structures was conducted employing the protein preparation module of the Schrödinger^34,35^. During this process, all components except the structure of interest were omitted. After that, hydrogen atoms were added to the structure, and water molecules were removed from the structure. The system was then minimized via the OPLS3e force field. The PIPER of Schrödinger was employed to calculate the interaction energy between the built NBs and CD20. This software considers one of the proteins as the receptor and the other one as the ligand. It does not matter which one is the receptor or ligand for the software. The number of ligand rotations to probe was set to 70,000, and no constraints were applied to the system. The PIPER is based on the Fast Fourier Transform (FFT) correlation method and can evaluate a vast number of conformations^36^. Utilizing this approach reduces the number of initial poses that require extra rigid-body filters and computationally costly electrostatic computations. This method could assess up to 70,000 distinct poses of the protein–protein complex. Eventually, PIPER presents the top-ranked poses based on the energies.

### Rational mutation protocol

The potential amino acids that can affect the binding affinity between the NB and the target have to be determined. Therefore, the contact distance was considered an adequate criterion^37^. The molecular docking of the original NB against CD20 was accomplished, and the contact distances of the residues were monitored. The residues with a contact distance of less than 2.2 Å were selected for the mutation. There are a large number of NBs that could be constructed by combining the selected residues. There should be a proper and trustworthy selection of amino acids for the preparation of NBs, since there is a massive number of possible mutations. Therefore, the experimental design was employed to construct a limited number of mutated NBs. DOE can reduce the number of experiments rationally. DOE was performed by the Taguchi method^38,39^. The binding energies of the designed NBs were calculated, and the results were interpreted by the software (MINITAB17). It suggests the key mutations and can statistically evaluate the parameters. The experimental design results can introduce the best set of mutated residues to build the NB with the highest binding affinity^40^. There were six residues with a contact distance less than 2.2 Å. These residues should be replaced with a rational mutation. The amino acids for replacement were Trp, Phe, His, Asp, and Tyr^41^, which could enhance the binding affinity of NBs. The selected arrays of these amino acids in the position of the key residues were set by the experimental design (Taguchi method). The arrays suggested by DOE are presented in Table 1. After evaluation of the binding affinities of the constructed NBs, the results were assessed and the proper amino acid for each position was determined.

**Table1:** The residues with a contact distance less than 2.2 Å, the amino acids suggested by DOE for mutation, and the calculated binding energies as the results.

No	Res27	Res28	Res29	Res31	Res99	Res100	Binding energy (kcal/mol)
1	Trp	Trp	Trp	Trp	Trp	Trp	− 750
2	Trp	Phe	Phe	Phe	Phe	Phe	− 717
3	Trp	His	His	His	His	His	− 677
4	Trp	Asp	Asp	Asp	Asp	Asp	− 650
5	Trp	Tyr	Tyr	Tyr	Tyr	Tyr	− 735
6	Phe	Trp	Phe	His	Asp	Tyr	− 705
7	Phe	Phe	His	Asp	Tyr	Trp	− 677
8	Phe	His	Asp	Tyr	Trp	Phe	− 692
9	Phe	Asp	Tyr	Trp	Phe	His	− 791
10	Phe	Tyr	Trp	Phe	His	Asp	− 683
11	His	Trp	His	Tyr	Phe	Asp	− 672
12	His	Phe	Asp	Trp	His	Tyr	− 701
13	His	His	Tyr	Phe	Asp	Trp	− 737
14	His	Asp	Trp	His	Tyr	Phe	− 673
15	His	Tyr	Phe	Asp	Trp	His	− 689
16	Asp	Trp	Asp	Phe	Tyr	His	− 735
17	Asp	Phe	Tyr	His	Trp	Asp	− 656
18	Asp	His	Trp	Asp	Phe	Tyr	− 673
19	Asp	Asp	Phe	Tyr	His	Trp	− 686
20	Asp	Tyr	His	Trp	Asp	Phe	− 701
21	Tyr	Trp	Tyr	Asp	His	Phe	− 686
22	Tyr	Phe	Trp	Tyr	Asp	His	− 741
23	Tyr	His	Phe	Trp	Tyr	Asp	− 675
24	Tyr	Asp	His	Phe	Trp	Tyr	− 675
25	Tyr	Tyr	Asp	His	Phe	Trp	− 740

### Determination of physicochemical properties and prediction of solubility

Various physicochemical properties of the designed NB, including MW, theoretical pI, instability index, aliphatic index, estimated half-life, and grand average of hydropathicity (GRAVY), were determined by the ProtParam tool (https://web.expasy.org/protparam/). The solubility of NB was also evaluated using the PROSO II server (http://mbiljj45.bio.med.uni-muenchen.de:8888/prosoII/prosoII.seam).

### Prediction of allergenicity and immunogenicity

The allergenicity of the designed NB was predicted using Algpred (https://webs.iiitd.edu.in/ragha va/algpred/submission.html). The sequence of the NB was uploaded into the server, and the allergen prediction was accomplished. Immunogenicity of the NB was also evaluated by VaxiJen 2.0 server (http://www.ddg-pharmfac.net/vaxijen/VaxiJen/VaxiJen.html).

### Molecular dynamics (MD) simulation

The interactions constructed between the NB and CD20 have to be further assessed dynamically. MD simulation is a valuable strategy that can assess the binding characteristics reliably. Desmond of Schrödinger^42^ was employed to perform the MD simulation. The optimized NB with the lowest binding energy (the highest binding affinity to CD20) was evaluated by performing the MD simulation. The optimized NB was aligned with CD20, and the system was minimized employing desmond. The MD simulation was conducted in an orthorhombic box, and the solvent model of transferable intermolecular potential with 3 points (TIP3P) was selected for the simulation^43^. The appropriate number of Na + /Cl − ions with a salt concentration of 0.15 M were employed to neutralize the system using the system setup of Schrödinger^44^. The prepared system was then simulated for 100 ns with the default relaxation protocol of software and the constant number of atoms, pressure, and temperature (NPT) ensemble^45^. The Nose–Hoover protocol was used to set the temperature to 310.15 K (37 °C), and the pressure was adjusted to 1 atm employing isotropic scaling^46^. For a better comparison, MD simulation of the original NB was also carried out.

## Results

### Homology modeling and molecular docking

**Figure 2:**
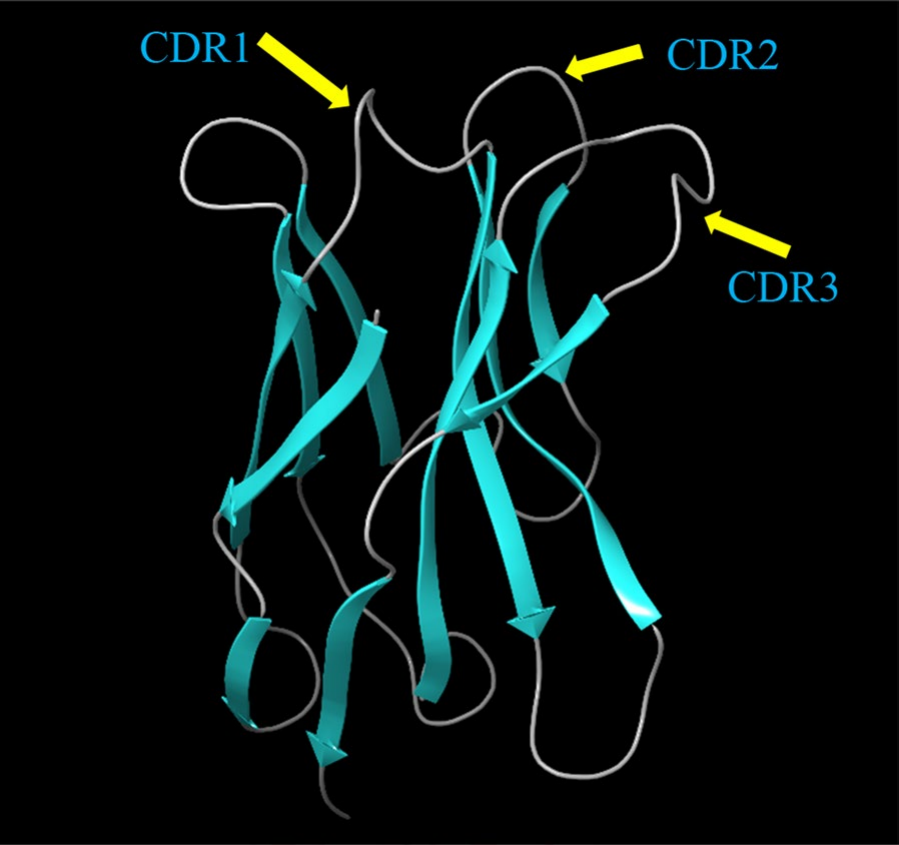
3D structure of the original NB (patent number: US20180079822) built using the templates 6C5W and 5JQH. Visualization was done using the PyMol Molecular graphic system (Schrödinger, LLC), https://pymol.org/2/.

Based on the BLAST homology search results, two structures, namely 6C5W and 5JQH, were selected as proper templates. The sequence alignment of the NB and two structures is presented in Fig. 1A. The structure of the built NB was evaluated by the Ramachandran plot (Fig. 1B). As can be seen, the majority of the residues are placed in the most favored regions. The 3D structure of the NB is shown in Fig. 2. The binding characteristics of the NB and CD20 were then investigated using molecular docking. The outcomes obtained from the docking study revealed that six residues had a contact distance of less than 2.2 Å (supplementary information, Table S1). These residues were Ser27, Thr28, Phe29, Ile31, Asp99, and Asn100. Interestingly, these residues are from CDRs of the NB. Residues 27, 28, 29, and 31 belong to CDR1, and residues 99 and 100 belong to CDR3.

### Rational mutation and affinity maturation

**Figure 3:**
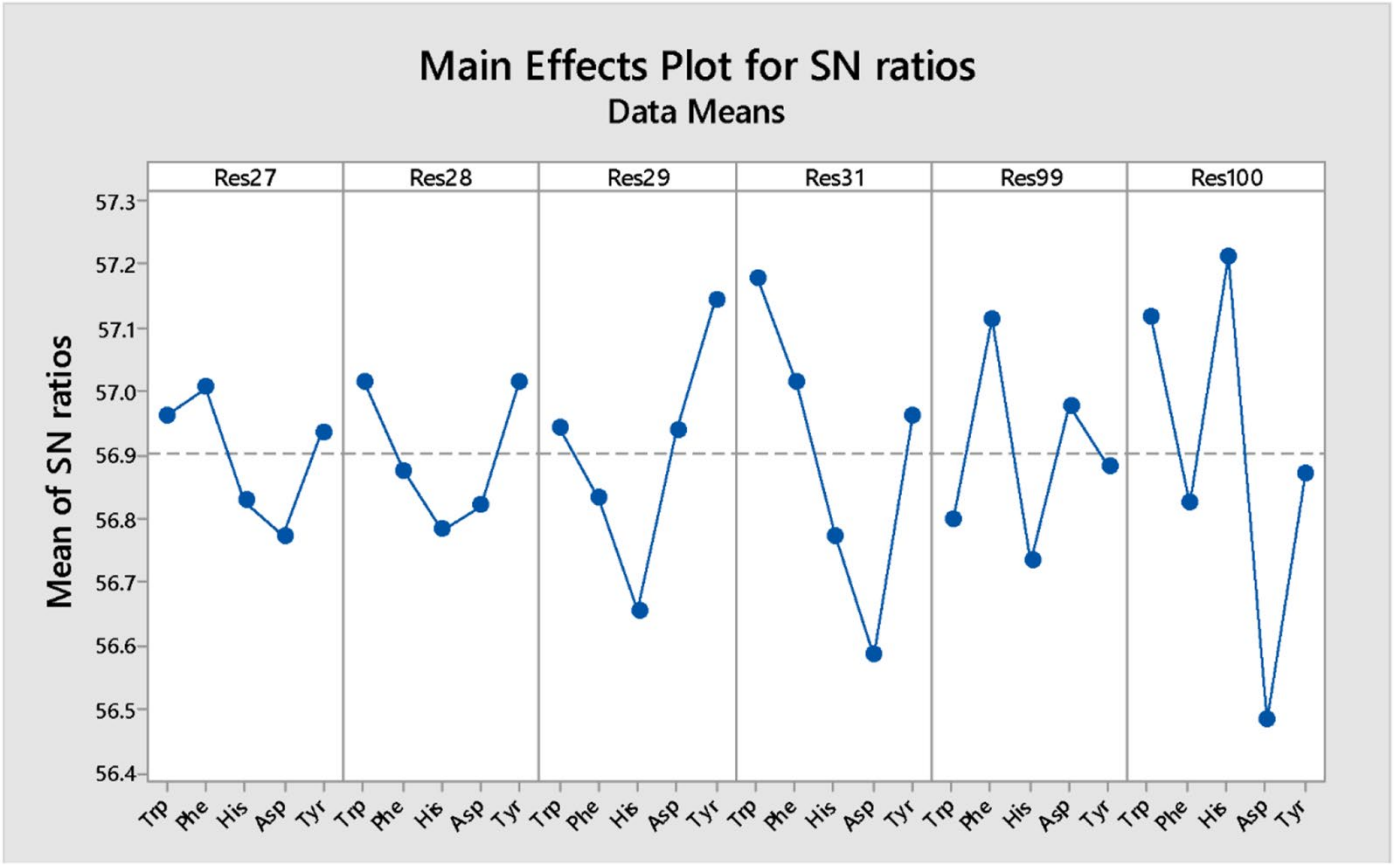
The main effects plot of various arrangements of the selected residues after mutation by the favorite amino acids.

**Figure 4:**
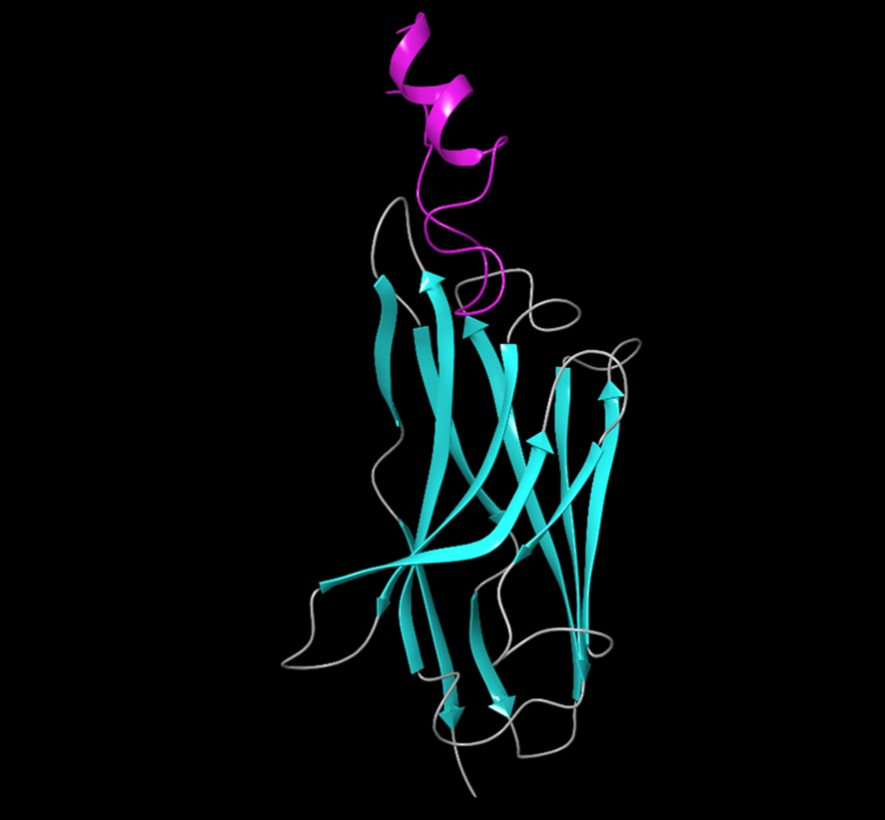
The docking pose of NB2 (cyan) and CD20 (purple). The visualization was done using the PyMol Molecular graphic system (Schrödinger, LLC), https://pymol.org/2/.

Based on the results obtained from the docking study, six candidate amino acids should be considered for the mutation. The outcomes also revealed that the amino acids are from the CDR regions of the NB. Since the CDRs are loops, the amino acids that are suitable for loops should be selected. In a study accomplished by Gavenonis et al., the amino acids were categorized based on their suitability for being used in the loops^41^. Their study introduced the amino acids that could improve the protein–protein interaction based on binding energy. Therefore, the top-ranked amino acids were considered for mutation. These amino acids were Trp, Phe, His, Asp, and Tyr.Obviously, mutation of six amino acids and replacing them with five amino acids would result in a large number of mutated structures. The number of mutated structures is 15,625, which is a vast number. The number of experiments can be rationally decreased by employing experimental design. For this purpose, the Taguchi method was employed, and the MINITAB software was used to perform DOE^40^. This method defines the experiments and gains the result to introduce the best set of amino acids. The results were the binding energies between NBs and CD20. The suggested experiments and the calculated binding energies are displayed in Table 1. The binding energy of the original NB was − 745.6 kcal/mol. The software gained the binding energies of the mutated NBs and analyzed them. The results were interpreted by the software, and for each one of the residues (residues 27, 28, 29, 31, 99, and 100), the selected amino acids had a different impact on the binding affinity. The favorite amino acids for the selected residues were determined by DOE, which is presented in Fig. 3 (The main effect plot for the signal-to-noise ratios). The higher mean of SN ratio indicates a greater positive impact on the desired result (lower binding energy). For instance, phenylalanine was predicted to be the best amino acid for residue 27. For residues 29, 31, 99, and 100, the best amino acids were tyrosine, tryptophan, phenylalanine, and histidine, respectively. As shown, there were two suitable amino acids for residue 28, namely tryptophan and tyrosine. Therefore, two NBs were constructed: NB1 (res27: Phe, res28: Tyr, res29: Tyr, res31: Trp, res99: Phe, and res100: His) and NB2 (res27: Phe, res28: Trp, res29: Tyr, res31: Trp, res99: Phe, and res100: His). The binding affinity of these best NBs was evaluated using molecular docking calculations, and the binding energies were obtained. The binding energy of NB1 was about − 794 kcal/mol, and this value was − 853 kcal/mol for NB2. These binding energies showed that NB2 had a more remarkable binding affinity to CD20 and the predicted results of DOE were accurate and reliable. Considering the binding energy of the original NB (− 745.6 kcal/mol) and other designed NBs (Table 1), the binding energy of NB2 (− 853 kcal/mol) indicated the ability of this newly designed NB as a potential inhibitor of CD20. The docking pose of NB2 and CD20 is presented in Fig. 4, and Table 2 shows the details of the docking results.

**Table 2:** The binding site residues of the optimized NB2 and the closest residues of CD20 with their contact distances.

Residue of NB2	Closest residues in CD20	Contact distance (Å)
Asp 1	Tyr 182	2.2
	Ile 186	2.6
	Ser 185	3.5
Val 2	Ile 186	2.7
Gly 26	Ile 186	1.1
Phe 27	Gln 187	0.7
	Tyr 165	1.6
	Cys 167	2.7
	Cys 183	2.8
	Ile 186	3.2
Trp 28	Tyr 165	0.8
Tyr 29	Tyr 165	3.1
Trp 31	Ile 164	1.1
	Tyr 165	3.0
	Asn 166	3.4
Lys 32	Tyr 165	3.7
His 33	Ile 164	2.6
Phe 99	Gln 187	2.1
	Ile 186	3.0
		3.4
His 100	Ile 164	1.5
	Tyr 165	1.5
	Gln 187	2.4
	Asn 166	2.5
	Cys 167	3.3
Asp 101	Tyr 184	1.9
	Gln 187	3.5
Tyr 102	Tyr 184	1.9
	Glu 168	3.8
Leu 105	Ile 164	3.6
His 106	Nma 187A	1.7
	Gln 187	2.3
Arg 107	Nma 187A	2.6
Phe 108	Ile 186	2.1
	Gln 187	2.3
	Nma 187A	2.4

### Physiochemical properties and solubility

The MW of the optimized NB2 was about 13.4 kDa with a theoretical isoelectric point (pI) of 9.26, which indicated its alkaline nature. The estimated half-life of the NB2 was predicted to be 11.1 h in mammalian reticulocytes, in vitro and more than 10 h in *Escherichia coli, *in vivo. The instability index (II) of the NB was 24.69, which shows the NB2 is remarkably stable. The GRAVY was − 0.395, which indicates the hydrophilicity of NB2, and means it can interact with water molecules. The total number of negatively charged (Asp + Glu) and positively charged (Arg + Lys) residues were 8 and 12, respectively.

### Allergenicity and immunogenicity

NB2 was predicted to be non-allergenic by Algpred. The antigenicity score of the NB2 was predicted to be 0.61 by VaxiJen v2.0, which means the NB2 is highly antigenic.

### MD simulation

**Figure 5:**
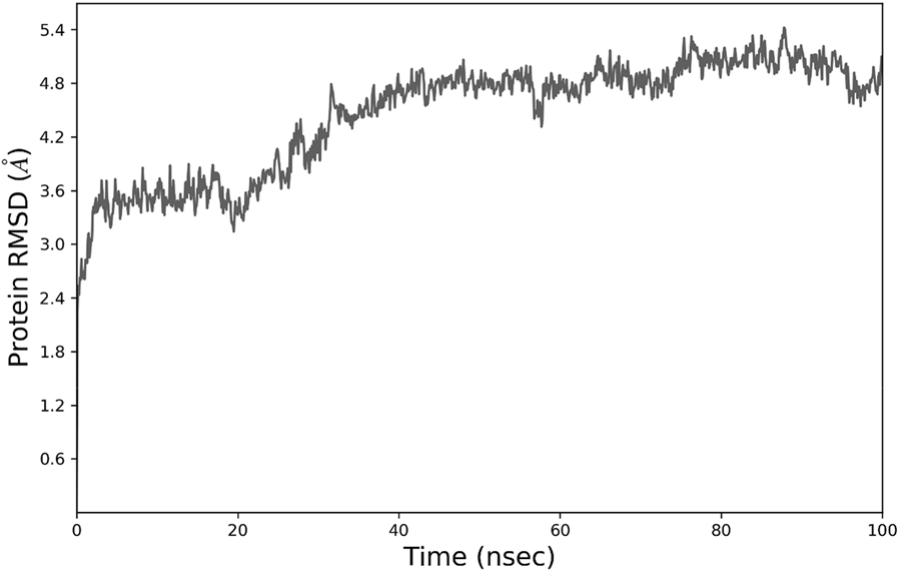
The RMSD of the NB2. The fluctuations were damped on about 4.8 Å.

**Figure 6:**
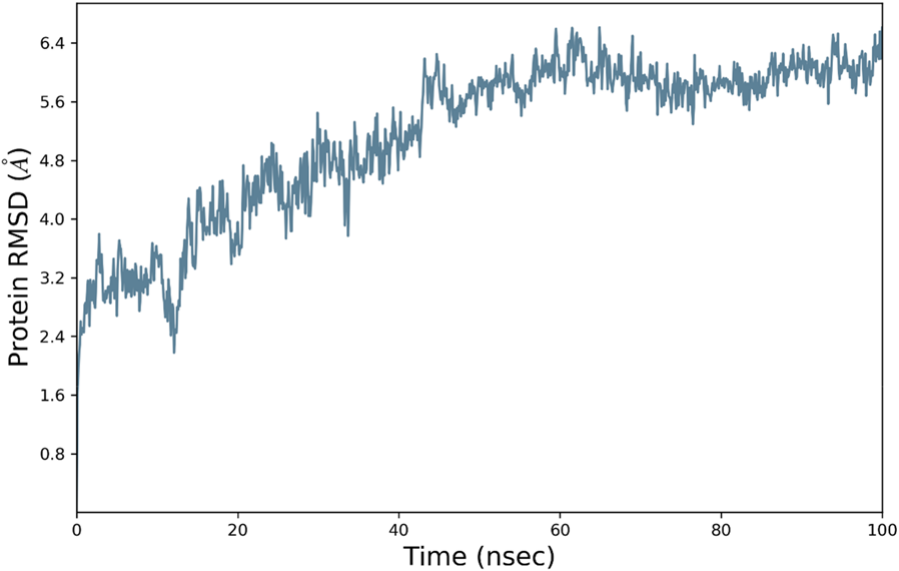
The RMSD of the original NB. The fluctuations are damped on about 6 Å.

**Figure 7:**
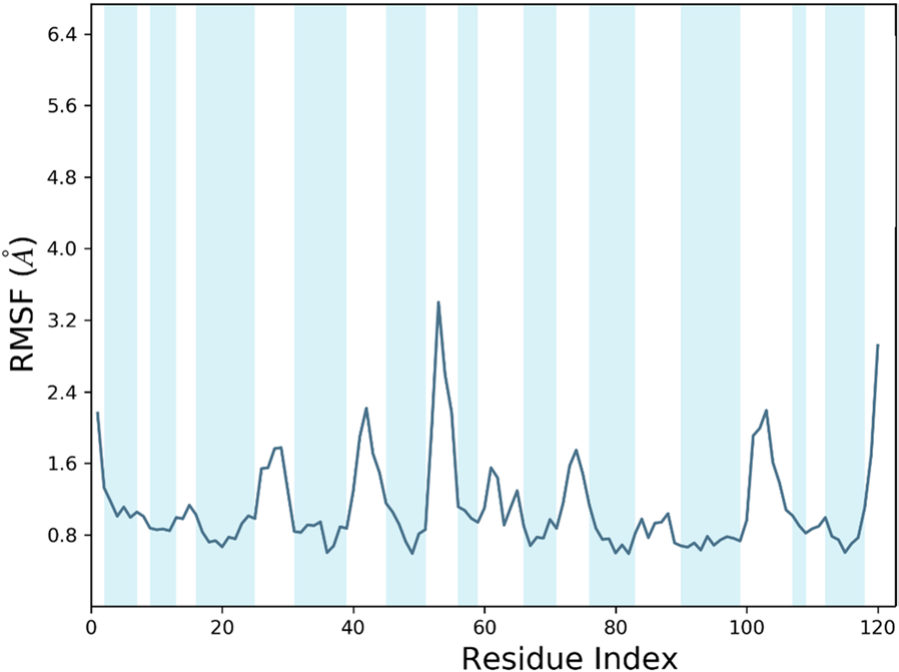
The RMSF plot of NB2 during the simulation. The blue regions are beta-strands, and the white areas show loops.

**Figure 8:**
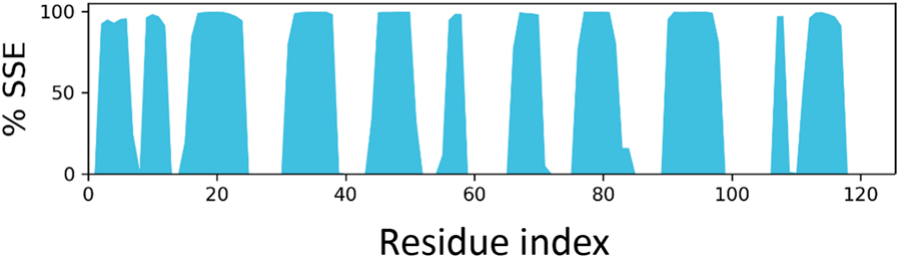
The secondary structure elements of NB2 during the simulation. Blue regions show the beta strands parts of the NB.

**Figure 9:**
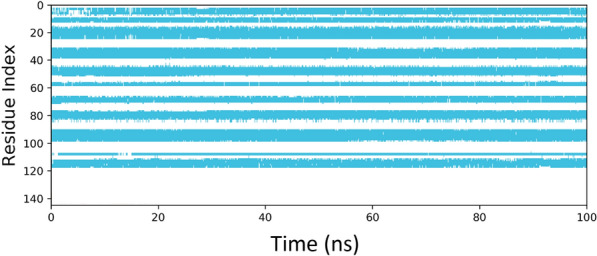
The trajectory parameters of PO5 during the fluctuation time.

MD simulation was conducted to better understand the NB's behavior in a dynamic condition. It can help to estimate the characteristics of the NB2 dynamically. The RMSD plot of the simulations showed the system reached equilibration after about 30 ns. Figure 5 shows the RMSD of NB2 after 100 ns, and the system was equilibrated at about 4.8 Å. The RMSD plot of the original NB is presented in Fig. 6. As could be seen, the fluctuations were stabilized at about 6 Å. Moreover, the original NB was equilibrated after almost 50 ns. The value of RMSD and the time of equilibration indicated that NB2 is more stable than the original NB. Another valuable outcome of the MD simulation is the RMSF plot, which indicated the stability of the residues during the simulation. Figure 7 represents the RMSF of the NB2 during 100 ns. As was expected, the unstructured parts of the NB, such as loops (white regions), had more fluctuations than beta strands (blue regions). As can be seen in this figure, the CDR1 (residues 26–33) and CDR3 (residues 96–108) are more stable than CDR2 (residues 51–57). Therefore, CDR1 and CDR3 play key roles in the recognition of CD20 by the NB2. The protein secondary structure element (SSE) is displayed in Fig. 8. The beta-strand parts of the NB are presented in blue. Figure 9 shows the SSE assignment over the simulation time.

## Discussion

Since 1997 by combination therapy of rituximab plus cyclophosphamide, doxorubicin, vincristine, and prednisone (R-CHOP), the percent of cured patients with diffuse large B-cell lymphoma has reached about 60–65%^47^. CD20 is an adequate target on the cell surface of the B-cells^48^, which could be targeted by mAbs. Rituximab is an approved therapy for patients with NHL that can specifically target B cells and induce reversible B cell depletion^49^.

Despite the satisfactory results achieved by rituximab, there are also some reports about the unsuccessful outcomes of this antibody^6^. The failure has several reasons, such as the high MW of the antibody, which can reduce tumor penetration and decrease its effectiveness^8^. Considering the restricted application of antibodies in some fields, NBs could be regarded as a promising alternative for them^50^. The small size, high stability, and remarkable specificity of NBs make them promising candidates for biological applications^51^. One of the most attractive uses of NBs is cancer therapy^52^, which could be applied to various cancer cells. A promising method for cancer therapy is cancer immunotherapy, which has attracted considerable attention in recent years^53^. Some studies reported NB-based immunotherapy for B-cell acute lymphoblastic leukemia, which shows the tremendous ability of NBs for cancer immunotherapy^54^. CD20 is a phosphoprotein expressed on the surface of all mature B-cells^55^ and overexpressed on the cell surface of B-cells NHL^56^. Targeting CD20 is an appropriate method for treating NHL^57^. Here, an optimized NB was computationally designed, modeled, and evaluated to construct a proper inhibitor against CD20. The 3D structure of the NB was constructed based on the optimal templates (6C5W and 5JQH), obtained by performing the BLAST homology search of BioLuminate. The interaction of the NB and CD20 was monitored by conducting molecular docking calculations. The residues with the minimum contact distance (cut off: 2.2 Å) were considered key residues for NB-antigen interactions. These residues were Ser27, Thr28, Phe29, Ile31, Asp99, and Asn100, which are from the CDR1 and CDR3 of the NB. Then, rational mutations were applied to these residues utilizing suitable amino acids. The amino acids with adequate characteristics for being put in the loops were selected based on the previous reports. These amino acids were Trp, Phe, His, Asp, and Tyr, which were reported to provide effective protein–protein interactions^41^. As there are many mutations, DOE was used to perform a certain number of mutations. This method obtains the results of the docking calculations (binding energies) and determines the best amino acid for each residue position. Therefore, the optimized NB could be easily constructed based on the experimental design results. The Taguchi method was used for DOE. The final residues for creating the optimized NB were res27: Phe, res28: Trp, res29: Tyr, res31: Trp, res99: Phe, and res100: His. The optimized NB (NB2) showed significant binding affinity to CD20 (− 853 kcal/mol). The characteristics of NB2 were further evaluated by MD simulation. The RMSD results showed that the system reached equilibration at 4.8 Å after 30 ns simulation. This value was 6 Å for the original NB, which occurred after 50 ns. It shows that NB2 is more stable than the original NB. The RMSF of the NB revealed that CDR1 and CDR3 are the critical loops in antigen recognition and binding to CD20. The protein SSE results indicated that the secondary structure of the NB was changed significantly.

Although in silico investigations have supported the potential activity of NB2 as an inhibitor of CD20, the outcomes should be experimentally evaluated. This optimized NB needs to be constructed in the wet lab, and its activity should be assessed experimentally.

## Conclusions

Although the current treatments for B-cells NHL, such as mAbs, have shown some failure in several cases, there could be practical approaches to overcome the limitations of traditional therapies. NBs are promising alternatives for mAbs with a lower MW and high binding specificity.They are suggested as applicable agents for biological purposes due to their stability and ability to recognize the antigens. NBs could be used for cancer treatment in various cancers, including NHL. CD20 is an appropriate target in NHL, which is highly expressed on the cell surface of malignant B cells. These NBs could target the membrane protein.

In this study, using computational methods, an optimized NB was designed by rational mutations in the CDR1 and CDR3. The rational mutations were suggested by experimental design, using the Taguchi method. The designed NB with the suggested mutations showed a remarkable binding affinity to CD20 in silico. Therefore, the optimized NB2 could be considered a potential inhibitor candidate against CD20. However, many further experimental investigations are needed to confirm its potency and usability in treating B-NHL.

This protocol is a practical approach for providing a certain number of mutations and determining the favorite mutations. Applying this protocol could create a new NB with a high affinity to the antigen. This procedure is especially beneficial when many mutations are induced, as a large number of experiments could be easily constructed and evaluated.

## Supplementary Information

Supplementary Information.

## Data Availability

The sequences generated and analyzed during the presented study are available from the Integrated Nanobody Database for Immunoinformatics (INDI) repository under the accession no. US20180079822.
