# Supplementary material for: Modeling and affinity maturation of an anti-CD20 nanobody: a comprehensive in-silico investigation
Source: Sci Rep. 2023 Jan 11;13:582. doi: 10.1038/s41598-023-27926-4 (PMC9834265; doi:10.1038/s41598-023-27926-4)
Supplement: Supplementary file 1 — Supplementary Information. [file 41598_2023_27926_MOESM1_ESM.doc]

***Supporting Information***

**Modeling and affinity maturation of an anti-CD20 nanobody: A comprehensive in-silico investigation**

**Alireza Poustforoosh^1,2*^, Sanaz Faramarz^3^, Manica Negahdaripour^4,5^, Hassan Hashemipour^6^**

1. Department of Chemical Engineering, Faculty of Engineering, Shahid Bahonar University of Kerman, Kerman, Iran
2. Medicinal and Natural Products Chemistry Research Center, Shiraz University of Medical Sciences, Shiraz, Iran
3. Department of Clinical Biochemistry, Afzalipour School of Medicine, Kerman University of Medical Sciences, Kerman, Iran
4. Department of Pharmaceutical Biotechnology, School of Pharmacy, [Shiraz University of Medical Sciences](https://scholar.google.com/citations?view_op=view_org&hl=en&org=11788014889566764788), Shiraz, Iran
5. Pharmaceutical Sciences Research Center, School of Pharmacy, Shiraz University of Medical Sciences, Shiraz, Iran
6. Chemical Engineering Department, Faculty of Engineering, Vali-e-Asr University of Rafsanjan, Rafsanjan, Iran

*** Corresponding author:**

Alireza Poustforoosh, Chemical Engineering Department, Faculty of Engineering, Shahid Bahonar University of Kerman, Kerman, Iran. Email: [alireza110_p@yahoo.com](mailto:alireza110_p@yahoo.com)

Table S1. The distances between the residues of NB and CD20 obtained from the molecular docking investigation.

| Residue of NB | Closest residue in CD20 | Distance |
| --- | --- | --- |
| A:4:Leu | P:163:Ace | 3.4 A |
| A:24:Ala | P:163:Ace | 3.1 A |
| A:27:Ser | P:164:Ile P:165:Tyr | 0.8 A 3.0 A |
| A:28:Thr | P:184:Tyr P:164:Ile P:167:Cys P:166:Asn P:165:Tyr | 2.1 A 3.2 A 3.3 A 3.5 A 3.5 A |
| A:29:Phe | P:187:Gln P:164:Ile P:167:Cys P:184:Tyr P:165:Tyr | 1.5 A 2.1 A 2.8 A 2.9 A 3.1 A |
| A:30:Ser | P:187A:Nma | 3.6 A |
| A:31:Ile | P:163:Ace P:164:Ile P:165:Tyr | 1.4 A 1.9 A 3.0 A |
| A:32:Lys | P:187A:Nma P:187:Gln P:186:Ile | 2.3 A 2.6 A 2.6 A |
| A:34:His | P:163:Ace | 3.6 A |
| A:76:Asn | P:164:Ile | 2.7 A |
| A:97:Ala | P:163:Ace | 2.8 A |
| A:99:Asp | P:165:Tyr P:187:Gln | 1.0 A 2.2 A |
| A:100:Asn | P:186:Ile P:187:Gln | 2.1 A 3.7 A |
| A:104:Phe | P:186:Ile P:165:Tyr P:183:Cys P:182:Tyr | 2.4 A 2.5 A 2.6 A 3.3 A |
| A:105:Leu | P:165:Tyr | 2.3 A |
| A:106:His | P:165:Tyr | 3.5 A |
| A:108:Phe | P:165:Tyr | 3.2 A |
